# Prevalence of Obesity and Associated Risk Factors Among Adolescents in Ankara, Turkey

**DOI:** 10.4274/Jcrpe.714

**Published:** 2012-12-19

**Authors:** Sırma Ercan, Yıldız Bilge Dallar, Serdar Önen, Özlem Engiz

**Affiliations:** 1 Ankara Training and Research Hospital, Department of Pediatrics, Ankara, Turkey; 2 Ankara Training and Research Hospital, Department of Pediatric Endocrinology, Ankara, Turkey

**Keywords:** adolescents, obesity, prevalence, risk factors, Turkish

## Abstract

**Objective:** The purpose of this study was to investigate the prevalence of and the risk factors associated with obesity among adolescents in Ankara, Turkey.

**Methods:** The study was conducted in 26 schools in Ankara during the time period from September 2010 to March 2011. A total of 8848 adolescents aged 11-18 years were chosen using a population-based stratified cluster sampling method. Body mass index (BMI) of the participants was compared with the BMI references for Turkish children and adolescents to estimate the prevalence of overweight and obesity. A standardized questionnaire aiming to determine the sociodemographic characteristics, computer use, television (TV) watching, physical activity, and presence of obesity in the family was applied to the study group.

**Results:** The results showed that the overall prevalence of obesity among adolescents was 7.7% (8.4 % for females and 7.0% for males). It was observed that BMI increased as computer use increased. A greater proportion of the overweight and obese adolescents watched TV and use computer for more than 2 hours/day as compared to their normal-weight counterparts. The normal-weight subjects were found to show a higher participation in regular physical activity. Obesity prevalence among the families of obese adolescents was 56.5%.

**Conclusions:** The prevalence of adolescent obesity in Ankara, Turkey is lower as compared to many European countries and to the United States. Computer use, watching TV, physical activity and family factors are important risk factors for obesity.

**Conflict of interest:**None declared.

## INTRODUCTION

The prevalence of childhood obesity is on the rise around the world. At present, more than 30% of children and adolescents 2-19 years of age are classified as overweight or obese with a body mass index (BMI) higher than the 85th percentile of the Centers for Disease Control and Prevention growth charts for the year 2000 (1,2). The World Health Organization (WHO) has designated obesity as a major public health problem in 1998 (3). Childhood and adolescence obesity is related to an increased adult morbidity and mortality by leading to a variety of conditions such as diabetes mellitus, hypertension, psychological disorders and social problems (4,5,6). Therefore, it is important to monitor overweight and obesity in children and adolescents.

Several studies in Turkey have reported associations of obesity with different risk factors. Discigil et al (7) found high socio-economic status to be associated with childhood obesity. Oztora et al (8) observed that obesity correlated with television (TV) viewing and computer use for more than 4 hours. Akac et al (9) reported a statistically significant increase in the frequency of risk factors such as unhealthy eating habits, spending long hours watching TV, family history of obesity and high socio-economic status in obese children. Studies on obesity prevalence in certain age groups have been published in Turkey, but there is limited data regarding adolescents. Thus, this study aimed to identify the obesity prevalence and risk factors in an adolescent population in Ankara, Turkey.

## METHODS

The anthropometric survey was conducted in Ankara during the time period from September 2010 to March 2011. A multistage random cluster sampling technique was used. A total of 8848 students were randomly selected from 26 schools in 6 districts of Ankara. A standardized questionnaire was distributed to these students. Gender, age, physical activity, computer use, TV viewing, as well as weight status of parents and family history of obesity were included in the questionnaire. Regular physical activity was defined as activity performed at least 3 days a week, each episode lasting at least 60 minutes.

The body weight and height of the students were measured by the same person. Weight was measured using a portable electronic scale with the subject wearing light clothes and no shoes. Height was measured using a stadiometer with the subject’s shoes off, feet together, and head in the horizontal plane. The BMI was calculated using the formula: body weight (kg) / height squared (m2). The degree of obesity was defined using the reference data on Turkish children (10).

The data were analyzed using the Statistical Package for the Social Sciences (SPSS v. 11.5, Chicago, IL, United States). Continuous variables with normal distribution were expressed as mean ± standard deviation (SD) and compared using the analysis of variance (ANOVA) test. Categorical variables were expressed as frequencies and compared between the groups with Pearson’s chi-square test or Fisher’s exact test. A p-value of less than 0.05 was considered statistically significant.

This study was approved by the Ethics Committee of the Ankara Training and Research Hospital. 

## RESULTS

The sample representing the age groups between 11 and 18 years consisted of 8848 subjects [4408 girls (49.8%) and 4440 boys (50.2%)]. The mean age was 14.2±2.2 years (range 11-18). Of the 8848 adolescents, 52.4% were attending primary school (11-14 years of age) and 47.6% were attending secondary school (15-18 years of age), thus, the subjects were divided into two groups (11-14 and 15-18 age groups) to evaluate the effect of age on risk factors of obesity. The prevalence of short stature was 1.5%. The overall prevalence of obesity in this representative sample of adolescents in Ankara was 7.7%. The prevalence rates of obesity in girls and boys were 8.4% and 7%, respectively; the difference was statistically significant (p<0.001). The prevalence rates of obesity in the 11-14 age group and in the 15-18 age group were 5.9% and 9.6%, respectively; this difference was also statistically significant (p<0.001) (Table 1).

The overall prevalence of computer use was 80%, being 72.3% in girls and 87.3% in boys; the difference was statistically significant (p<0.001). The prevalence rates of computer use in the 11-14 age group and in the 15-18 age group were 74.5% and 85.7%, respectively; this difference was also statistically significant (p<0.001). The adolescents in the 15-18 age group used the computer for a period longer than 2 hours/day as compared to the adolescents in the 11-14 age group (p<0.001).

This study showed that BMI increased as computer use (hours/day) increased. The overweight and obese adolescents used the computer for periods longer than 2 hours/day compared to their normal-weight counterparts (p<0.001) (Table 2).

The prevalence of TV viewing for ≥2 hours/day was 52.4% among the adolescents, while 4.5% did not watch TV. A greater proportion of the girls were TV viewers as compared to the boys (p<0.001). A greater proportion of adolescents in the 15-18 age group watched TV for 2 hours/day or for longer periods as compared to the adolescents in the 11-14 age group. Obese and overweight adolescents watched TV for more than 2 hours/day compared to normal-weight subjects (p<0.001) (Table 2).

Physical activity was performed regularly by 30.7% of adolescents and irregularly - by 15.6%. More than half (53.7%) of the adolescents were not involved in any physical activity. The prevalence rates of regular physical activity in girls and boys were 19.5% and 41.8%, respectively; the difference was statistically significant (p<0.001). The prevalence rates of regular physical activity among adolescents in the 11-14 age group and in the 15-18 age group were 23.5% and 38.8%, respectively; the difference was also statistically significant (p<0.001). The normal-weight subjects were engaged in more regular physical activity compared to their overweight/obese counterparts (p<0.05) (Table 2).

The overall prevalence of familial obesity (obesity in first-degree family members - one or both parents, siblings) was 22.8% in this study. The prevalence of familial obesity was found to be as high as 56.5% among the obese adolescents,, significantly higher than among the normal-weight and overweight subjects (p<0.001) (Table 2). Maternal obesity prevalence rate was 29.7% while paternal obesity prevalence rate was 27.7% in the study population. The obesity prevalence rate in both parents and siblings was 26.3% and 12.1% respectively.

## DISCUSSION

Obesity is prevalent in the developed world. Data from the National Health and Nutrition Examination Survey (NHANES) 1988-1994 illustrate an obesity prevalence rate of 13.7% in the 6-11 age group and 11.5% in the 12-17 age group in the United States (11). Also, the NHANES 2003-2006 has reported an obesity prevalence rate of 16.3% in the 2-19 age group (12). In Europe, the highest obesity prevalence rates in school-aged children have been estimated in Spain and Portugal, while the lowest rates have been reported from Slovakia, France, Switzerland, and Iceland (13).

Various reports in Turkish children and adolescents indicate that the prevalence of overweight and obesity may change by regions, however, the overall figures are lower as compared to many European countries and to the US (14,15,16,17,18). In our study, the overweight prevalence rate was 8.3% (7.1% in the 11-14 age group and 9.7% in the 15-18 age group) and the obesity prevalence rate was 7.7% (5.9% in the 11-14 age group and 9.6% in the 15-18 age group). These results were comparable to other Turkish studies but lower than those reported for European countries and the United States.

Obesity and sedentary life style are closely related. In this study, 20.9% of the adolescents reported using computer for more than 2 hours/day. Similar to other published studies, boys spent more time than girls using computer (19,20). Computer use was more prevalent in obese and overweight children. In this study, nearly half (52.4%) of the adolescents reported watching 2 or more hours of TV per day. The proportion of obese and overweight adolescents watching TV for more than 2 hours/day was higher as compared to normal-weight ones. A study by Ozmert et al (21) reported similar results. Children with a BMI z-score >2 SD watched TV for longer periods than those with BMI z-scores < -2 SD.

Decreased physical activity is a serious risk factor for obesity (22,23). In our study, more than 50% of the adolescents did not participate in any physical activity; 30% of the adolescents reported performing some regular activity. Boys declared more regular activity than girls. These results are similar to data published by Agazzi et al (20). Adolescents with normal body weight reported more regular physical activity compared to their overweight and obese counterparts.

Having overweight parents has been shown to be a strong determinant of childhood obesity (24). A study by Burke et al (25) demonstrated the associations between child BMI and parental BMI. In this study, obesity in fathers was associated with a four-fold increase in the risk of obesity at the age of 18 years in both sons and daughters with an independent eight-fold increase in the risk for daughters if mothers were obese. In our study, 56% of the obese adolescents had an obese family member. The mean BMI of the adolescents increased as the parental BMI increased. In another study including 3306 children aged 5-7 years and their parents, the BMI of the children was found to be significantly correlated with the parental BMI. The children’s BMI showed closer associations with maternal than with paternal BMI (26). In the present study, maternal obesity rate was highest in the study population. This may be due to mothers having a greater influence on their children’s nutrition in our society. Further prospective and comprehensive studies are needed on this topic.

In conclusion, the obesity prevalence among Turkish adolescents in the city of Ankara is relatively low as compared to many European countries and to the US. Computer use and TV viewing as well as physical activity and family factors are important risk factors for obesity in Turkish adolescents. Healthy nutrition and regular physical activity should be promoted to prevent obesity.

## Figures and Tables

**Table 1:**
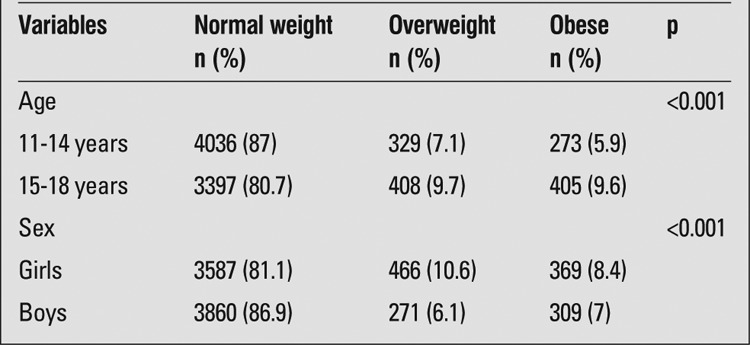
The distribution of obesity with respect to age and sex

**Table 2:**
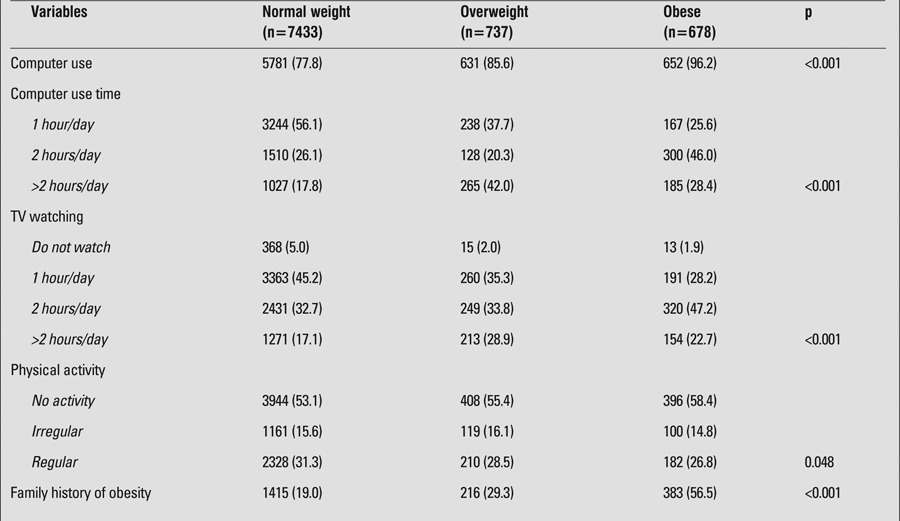
Relationships between obesity and computer use, television (TV) watching, physical activity and family history of obesity
